# Community-based participatory research and integrated knowledge translation: advancing the co-creation of knowledge

**DOI:** 10.1186/s13012-017-0696-3

**Published:** 2017-12-19

**Authors:** Janet Jull, Audrey Giles, Ian D. Graham

**Affiliations:** 1 Ottawa Hospital Research Institute, University of Ottawa, Ottawa, Ontario Canada; 2 School of Human Kinetics, Faculty of Health Sciences, University of Ottawa, Ottawa, Ontario Canada

**Keywords:** Community-based participatory research, Integrated knowledge translation, Engagement, Collaboration, Health systems, Co-creation, Knowledge, Implementation

## Abstract

**Background:**

Better use of research evidence (one form of “knowledge”) in health systems requires partnerships between researchers and those who contend with the real-world needs and constraints of health systems. Community-based participatory research (CBPR) and integrated knowledge translation (IKT) are research approaches that emphasize the importance of creating partnerships between researchers and the people for whom the research is ultimately meant to be of use (“knowledge users”). There exist poor understandings of the ways in which these approaches converge and diverge. Better understanding of the similarities and differences between CBPR and IKT will enable researchers to use these approaches appropriately and to leverage best practices and knowledge from each. The co-creation of knowledge conveys promise of significant social impacts, and further understandings of how to engage and involve knowledge users in research are needed.

**Main text:**

We examine the histories and traditions of CBPR and IKT, as well as their points of convergence and divergence. We critically evaluate the ways in which both have the potential to contribute to the development and integration of knowledge in health systems. As distinct research traditions, the underlying drivers and rationale for CBPR and IKT have similarities and differences across the areas of motivation, social location, and ethics; nevertheless, the practices of CBPR and IKT converge upon a common aim: the co-creation of knowledge that is the result of knowledge user and researcher expertise. We argue that while CBPR and IKT both have the potential to contribute evidence to implementation science and practices for collaborative research, clarity for the purpose of the research—social change or application—is a critical feature in the selection of an appropriate collaborative approach to build knowledge.

**Conclusion:**

CBPR and IKT bring distinct strengths to a common aim: to foster democratic processes in the co-creation of knowledge. As research approaches, they create opportunities to challenge assumptions about for whom, how, and what is defined as knowledge, and to develop and integrate research findings into health systems. When used appropriately, CBPR and IKT both have the potential to contribute to and advance implementation science about the conduct of collaborative health systems research.

## Background

Growing demands that are constrained by finite resources are raising concerns about the sustainability of health systems [1–3]. Research has a major role to play in ensuring that health care is sustainable, effective, efficient, safe, and appropriate. It is also widely accepted that society and health systems are not optimally benefiting from investments in research [4–6]. In particular, there remains a gap between what is known from research and the care that is provided in health systems. Reducing this “know–do gap” is one of the ethical imperatives of our time: there is a well-recognized need for implementation science [7] as the delayed implementation of effective practices (and discontinuation of ineffective ones) affects people’s health and contributes to the unsustainability of the health system. Better use of research evidence (one form of “knowledge”) in health care practice requires partnerships between those engaged in the processes that produce research and those who are contending with the real-world needs and constraints of health systems and their users.

One approach to bridging the know–do gap is to implement an interactive process of knowledge exchange between health researchers and knowledge users [8]. We refer to knowledge users here as individuals directly affected by research and inclusive of those who occupy a range of positions in health systems: funders, health system and policy decision-makers, health care providers, patients, and family members. Knowledge translation (KT), or the “knowledge to action” process, is defined by the Canadian Institute of Health Research (CIHR) [9] as:

a dynamic and iterative process that includes synthesis, dissemination, exchange and the ethically sound application of knowledge to improve the health of Canadians, provide more effective health services and products and strengthen the health care system. This process takes place within a complex system of interactions between researchers and knowledge users which may vary in intensity, complexity and level of engagement depending on the nature of the research and the findings as well as the needs of the particular knowledge user [para 4].

KT includes the tailoring of knowledge for use within specific contexts, and its use supports the development of evidence-based decisions. The primary purpose of KT is to bridge the know–do gap, ensuring that research is used by knowledge users such as government decision-makers and community service providers to improve health delivery systems and health outcomes [10]. That said, the conceptualization of the know–do gap as merely being the result of a knowledge transfer problem has been challenged. Current thinking has seen a shift to consider knowledge production as creating—or contributing—to the know–do gap [11, 12]. As a result, integrated knowledge translation (IKT) has been proposed as an approach to address the problematic issues with the generation of knowledge inherent in traditional research methods and knowledge production. Similarly, those who employ community-based participatory research (CBPR) have emphasized the importance of creating partnerships with the people for whom the research is ultimately meant to benefit. These research approaches engage researchers in partnerships with knowledge users and may be used to challenge assumptions about for whom, how, and what is defined as knowledge.

The generation of knowledge that can meet the needs of health systems’ knowledge users requires context-sensitive approaches and research structures; these can support the development and integration of what can be defined by those the knowledge is meant to benefit as best evidence. There is growing interest in collaborative approaches to knowledge generation between knowledge users and researchers and that lead to “co-created” knowledge [13]. The co-creation of knowledge in principle conveys the promise of significant social impacts, and the operations of knowledge user–researcher collaborations continue to be explored to advance the understandings of how to engage and involve knowledge users who deliver and/or receive care within health systems [14]. The evidence about how to best engage and involve knowledge users who are in health systems is being built [15, 16], and there are calls for consistency and systematic reporting to advance the field of collaborative research [17]; in addition, evidence indicates that participatory approaches to research show promise for increased levels of collaboration among community partners, researchers, and organizations [18] and the conduct of collaborative research [19, 20]. CBPR and IKT are approaches to research that make contributions to the practice and science of implementation research as they provide opportunities to advance understandings of processes and factors that facilitate and hinder the development and sharing of knowledge in health systems.

In this paper, we examine the histories and traditions from which CBPR and IKT have emerged, as well as their points of convergence and divergence. We critically examine the ways in which both have the potential to contribute to the development and integration of knowledge in health systems. Both IKT and CBPR have underlying drivers and rationales for their use that result in similarities and differences that span the areas of motivation, social location, and ethics. Nevertheless, the practices of CBPR and IKT converge upon a common aim: the co-creation of knowledge that is the result of both researcher and knowledge user expertise. We argue that CBPR and IKT can contribute evidence to implementation science and practices for collaborative research; however, clarity about the purpose for the research—social change or application—is a critical feature in the selection of an appropriate collaborative approach with which to build knowledge.

## Main text

### History/tradition of CBPR

There are various terms that have been used to describe iterations of CBPR, including but not limited to action research, participatory action research, and feminist participatory action research. Despite different terminology, these approaches share a commitment to working in partnership with members of marginalized communities to reduce or eliminate injustices and/or inequities that have been identified by community members themselves; further, those who use these approaches aim to create more equitable research processes between researchers and community members, particularly compared to more conventional research approaches [21]. For simplicity, we will use the term CBPR as an umbrella term for such approaches. The Kellog Foundation [22] defines CBPR as a “collaborative approach to research that equitably involves all partners in the research process and recognizes the unique strengths that each brings. CBPR begins with a research topic of importance to the community, has the aim of combining knowledge with action and achieving social change to improve health outcomes and eliminate health disparities” (para 2) [22].

Darroch and Giles [23] identified three main influences on CBPR: Kurt Lewin, Paulo Friere, and feminist theorists. Lewin [24] is credited with the term “action research”; he defined this approach as a research methodology in which members of communities are involved in every stage of the research process, including identifying the issue to be addressed, forming a plan, taking action, and then evaluating the outcomes. Friere’s work in education with non-literate Brazilians resulted in his famous 1970 text, *Pedagogy of the Oppressed* [25]. Friere rejected the notion of hierarchies in education, advocated for an equalization of relations of power, and encouraged the oppressed to examine their own oppression and then bring about social change, an approach deemed “participatory action research” [25]. He noted, “to liberate the oppressed without their reflective participation in the act of liberation is to treat them as objects that must be saved from a burning building” [25, p. 65]. He argued that we must treat members of marginalized communities as engaged subjects rather than objects; this requires their full participation in their liberation, and thus, their participation is needed throughout every stage of the research process. Feminist scholars, too, have played a major role in the development and articulation of strategies to increase the uptake of research findings. They have argued for the importance of including women in research, recognizing the home as a place of oppression, and treating women’s experience both inside and outside of the home as both political and worthy of study [26]. Further, feminist scholars have been strong proponents of conducting research “with” rather than research “on” individuals who experience socially structured disadvantage, and they have advocated for approaches that minimize hierarchies in research [27].

Within qualitative research, CBPR is defined as a methodology. Crotty defines a methodology as “the strategy, plan of action, process or design lying behind the choice and use of particular methods and linking the choice and use of methods to the desired outcomes” [28, p. 3]. A methodology can be contrasted with a method, which refers to “the techniques or procedures used to gather and analyze data related to some research question or hypothesis” [28, p. 3]. CBPR methodology enables researchers to use a wide variety of research methods, including but not limited to photo elicitation [29], focus groups [30], semi-structured interviews [31], participatory mapping [32], photovoice [33], and digital storytelling [34]. CBPR can also be used with a range of theoretical traditions [23], including, but not limited to, feminist theory, poststructural theory, and postcolonial theory [35].

Despite some differences in how CBPR is employed, one common aspect is a commitment to de-centered research “expertise.” Community members’ knowledge is viewed as legitimate and expert in nature [36]. Community members may be involved in every stage of the research process, from issue identification to the crafting of research questions; to research design, data collection, and analysis; and to writing and dissemination. The aim of CBPR is to emancipate participants and ultimately lead to social transformation [37]. For some projects, community members might delegate certain responsibilities (e.g., data analysis) to researchers but, ideally, they are involved in every aspect of the project. Involvement of knowledge users in research is gaining momentum in health systems research internationally [38]. These interests are reflected in the KT literature and, in particular, the literature of IKT.

### History/tradition of IKT

IKT is a research funder innovation, initially advanced by the Canadian Health Services Research Foundation as Knowledge Exchange [39] and more recently by the Canadian Institutes of Health Research as IKT. IKT involves a collaborative approach between researchers and knowledge users in the research process [40]. Although explicit mention of theory with IKT is rare [41], IKT is an approach to research and can be used with a range of theoretical research traditions such as the examples of IKT studies that used a biomedical [42] or postcolonial theory [43]. IKT also carries on the practice of knowledge user–researcher collaboration evident in CBPR, ideally in every step of the research process: development of the research question(s), decisions about methodology, involvement in data collection and tool development, interpretation of findings, and participation in the dissemination of findings [44]. Implicit in the IKT process is collaboration between researchers and knowledge users to address a research issue. Knowledge users and researchers in the partnership recognize that each member brings valuable insights and expertise and that knowledge ought to be generated collaboratively. Knowledge users typically have detailed knowledge of contextual and implementation factors, such as the strategic management of stakeholder relations (those who may have an impact or be effected by the knowledge) [45]. In turn, researchers bring expertise of research methods and methodologies. Knowledge users and researchers complement one another with the expertise brought to the research process.

IKT as a distinct research approach comes from a nascent body of work that has originated primarily from within the context of research in Canadian health systems to encourage collaborative research and is aimed at development of applicable evidence. A major funder of health research in Canada, CIHR, recognizes that IKT employs similar principles to that of CBPR, by bringing researchers and knowledge users into full partnership throughout the research process [44]. Although IKT does not have a long history as a distinct research approach, researchers are striving to define approaches to delineate, structure, and guide IKT research and implementation [46, 47]. An IKT approach has been referred to using a range of terms including collaborative research, action research, participatory research, coproduction of knowledge [48] or mode 2 research (i.e., working with end users) [40], and “engaged scholarship” [12]. Understandings of IKT and its related concepts continue to take shape as researchers, funders, policy analysts, and decision-makers, with community partners, increasingly look for new and innovative approaches to build knowledge that is applicable and thus more likely to effect health systems change. Such changes are enacted with full inclusion of knowledge users as partners who are viewed as integral to the process of knowledge creation.

### Points of divergence and convergence of CBPR and IKT

**Fig. 1:**
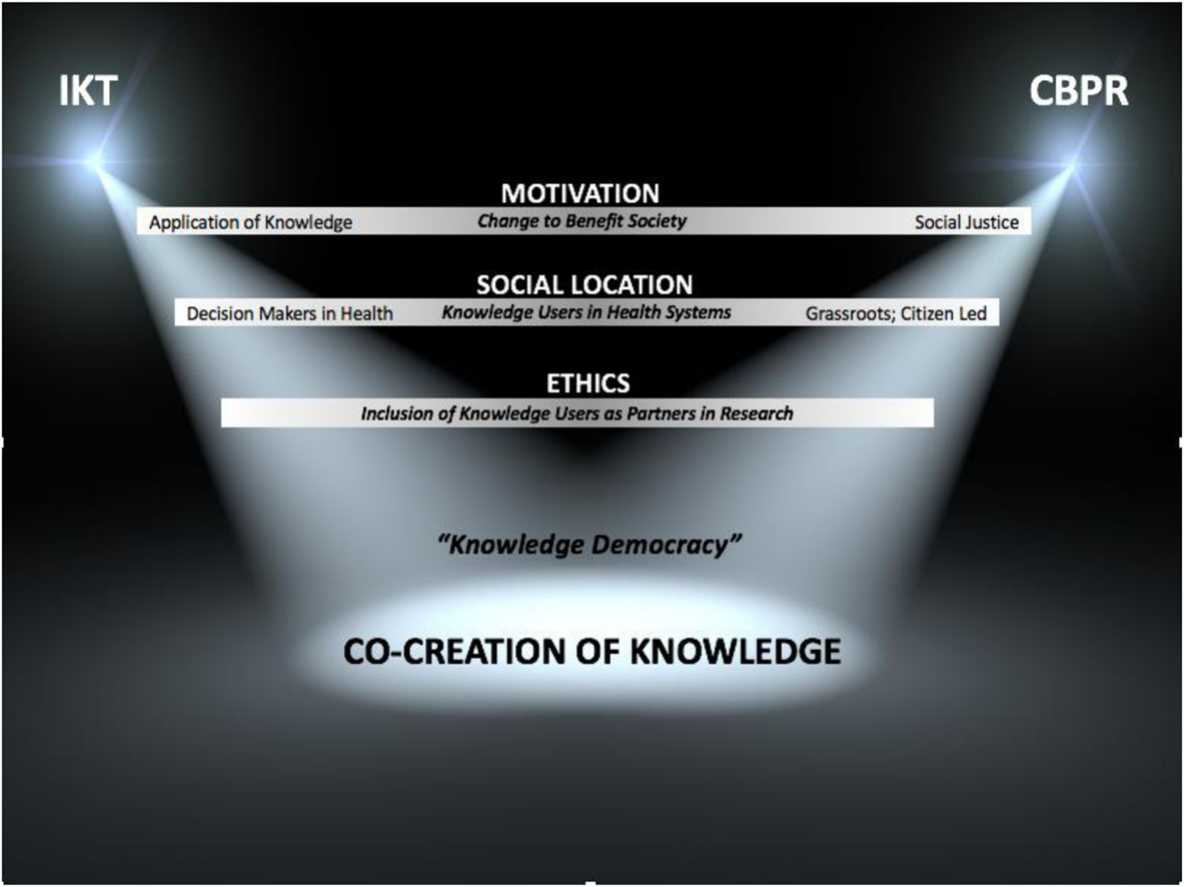
Points of divergence and convergence of IKT and CBPR

We critically examine the points of convergence and divergence for CBPR and IKT and identify that both CBPR and IKT have similarities and differences that span the areas of research motivation, social location, and ethics, important considerations in the conduct of collaborative research. As research endeavors, the practices of CBPR and IKT result in convergence upon a common aim: the co-creation of knowledge that is the result of knowledge user and researcher expertise (Fig. 1). CBPR and IKT are approaches to research that accommodate and facilitate the engagement and involvement of knowledge users with researchers, and they can both contribute evidence to existing implementation practices for collaborative research.

#### Motivation

Practitioners of CBPR and IKT differ in their motivations for the conduct of research. CBPR is underpinned by principles related to social justice and a desire for social change [24–26] such as the example of a knowledge user–researcher partnership to develop a faith-based educational intervention to promote cancer awareness in the African–American community that helped to define intervention impacts and address health disparities in underserved communities [49]. IKT practitioners’ focus is to promote research that is collaborative, addresses problems meaningful to the user of the research, and is most likely to develop applicable knowledge [40]. For example, a knowledge user–researcher partnership used an IKT approach to develop and evaluate a distance treatment program for child mental health that was transferred into clinical practice [50]. An IKT approach to research may aim to develop applicable knowledge that will have an impact on social justice, but it is the need for applicable knowledge, not social justice, that is the primary motivator for the research. While lacking an explicit and primary focus on social justice, IKT is based on a definition of KT that emphasizes “ethically sound application of knowledge to improve health, provide more effective health services and products, and strengthen the health care system” [40, para 1]. Social justice in health is defined as achieved with the highest attainable standard of physical and mental health [51]. While CBPR and IKT researchers may differ in their motivations, they have in common the aim to engage in research that will either enact social change (CBPR) or create applicable knowledge (IKT) and that serve to benefit society.

The differences and commonalities between CBPR and IKT are evident in the collaborative research ethos that knowledge users and researchers strive to create within a CBPR or IKT research partnership. CBPR and IKT researchers approach partnerships in research differently. Researchers have noted that IKT approaches create an opportunity for researchers and knowledge users to work together collaboratively and to make optimal utilization of the expertise that each brings to the partnership [52]. CBPR differs from IKT in that as a research approach, the aim of CBPR is to not only utilize the knowledge user expertise but also enhance their capacity for meaningful and equitable participation through the research, a critical feature of the approach [53]. The emphasis on knowledge user members’ capacity building evident in the CBPR [54] is not evident within IKT, where capacity building is centered on the research processes, that is, the engagement of researchers with knowledge users in the most effective creation of knowledge and its translation into action [55].

It has been suggested that the IKT process involves “a group level identity transformation” [52, p. 190] that allows for the fulsome incorporation of a range of perspectives that are inclusive of different areas of expertise. Such a point of view aligns well with the efforts of CBPR researchers who have also identified transformative processes within partnerships as a feature of the CBPR approach [56] and that are directed at the co-creation of knowledge. These views contrast with investigator-led research (that is, research that does not engage with knowledge users), and that may lack contextual relevance to the knowledge user setting and/or populations. Empirical evidence suggests that there has been a failure in moving research evidence into practice [8] and evaluation has identified the existence of a meaningful partnership (as defined by the researcher and knowledge users) as a catalyst for increasing both the relevance and use of research [20]. Collaborative research approaches such as CBPR and IKT may provide opportunities within health systems to better understand implementation science and practice to not only engage knowledge users in collaborative research but also complement investigator-led research that may more widely benefit health systems.

Health systems are complex and inter-related [57] and involve many different researcher and knowledge user perspectives, so it is important for collaborative research to clarify the motivations for research (i.e., driven by a desire for social change and/or a focus on developing evidence that is applicable to health systems). Thus, having both the IKT and CBPR literature to draw upon will be helpful for those who desire to learn about or who are involved in the implementation of collaborative research endeavors and/or who wish to accommodate a range of motivations to achieve particular health systems’ outcomes. For this reason, being clear on the motivation for research conduct is an important feature in the decision to use CBPR or IKT and is linked to the social location of the research approach.

#### Social location

CBPR and IKT originate from different social locations. CBPR originates in “grassroots” or citizen-led, democratic research traditions with attention to power relations [24–26], such as the example of a CBPR study that aimed to address heart health issues with women who were underserved [58]. IKT originates in research practices promoted by funders [39] and developed to engage with decision-makers who are knowledge users [59], as in the example of a study partnership formed with knowledge users (policy-makers and social workers) to identify priority health issues and to incorporate these into an effective training curriculum [60]. While CBPR and IKT research studies differ in their attention to power relations, they both describe knowledge user engagement practices [40, 61] and expand the recognition of who the knowledge users are and who may have the capacity for action on research outcomes [37, 52, 59] .

CBPR users seek to work in partnership to reduce or eliminate injustices and/or inequities that have been identified by knowledge users and through the enactment of equitable research partnerships in which sharing of knowledge and resources occurs between researcher and knowledge user partners as well as the broader community [62]. CBPR is intended to benefit those participating in the research and their communities through the research process and products. It is an approach to research in which the community exercises authority, that is, control and influence within the research, and this is a form of empowerment [63], a key principle in of CBPR [62]. IKT is also focused on research conduct to address issues identified as important by the knowledge users and the application of research results to ameliorate the identified health systems’ issue, but addressing power relations between those who will use or be impacted by the knowledge is not a primary aim [55]. Empowerment of participants to function within the IKT partnerships is identified as a key factor for achieving success in research [64]; however, while there may be power differentials to address, IKT did not originate to address power differentials within the conduct of research or the context of society.

In CBPR and IKT, researchers and knowledge users recognize and focus upon partnerships to drive a mutually agreed upon research agenda. As well, they are embedded within and have evolved from societies and movements that require governments to share in the processes of agenda-setting and the generation of policy [37, 65–68]. Both IKT and CBPR promote engagement of knowledge users in health systems’ research and contribute to the evidence on how, exactly, to conduct research with knowledge users within the health systems’ context [40, 44, 69, 70]. To do so requires investments in time and effort (including financial) to create opportunities for relationships and find common points of interest that are not yet typical in research endeavors [20, 71]. For both CBPR and IKT, research is done in ways that those within the collaboration agree are best to accomplish the aims of the research endeavor and which include consideration of ethics.

#### Ethics

The importance of engaging with knowledge users who have not typically been included in health systems research to improve knowledge development and dissemination is recognized in both the literatures of CBPR and IKT [52, 59, 65] as well as that of implementation science [8, 72, 73]. The inclusion of knowledge users as partners in research must be carefully considered in the design and conduct of research studies to ensure respect for forms of knowledge and knowledge systems that differ from that held by the researchers [74, 75]. All research designs are guided by ethical standards articulated in guidelines although they may not adequately reflect views on ethical conduct that resonate with and advance research agendas that are of value to knowledge users (that is, communities and their members) [71]. For example, in Canada, researchers who engage in research are prompted to consider how to conduct their work with individuals and within communities in mutually agreed upon ways [76]. There are under-acknowledged differences between standard, researcher-oriented research traditions and the beliefs, values, and cultural perspectives of knowledge users. These differences are believed to undermine opportunities for participation by knowledge users in research endeavors [77]. The use of CBPR or IKT research approaches that engage knowledge users as full and active partners with researcher partners can prompt thoughtful consideration of how to operationalize ethical conduct within research.

Knowledge user–researcher relationships in CBPR and IKT studies disrupt the division between those who do research and those who are participants in the research. CBPR and IKT provide examples of ways to conduct research that engages with a broad range of knowledge users in ways that knowledge users themselves can define as ethical and thereby acceptable [78, 79]. Importantly, collaborative research has been found to create opportunities for real change: involvement in research by knowledge users happens more often; the research is more likely to influence the behavior of knowledge user partners; and there is the creation of real-world applicable knowledge [20]. CBPR and IKT studies provide evidence of sharing information and expertise and examples of how to enact research relationships that foster ethical research partnerships and, ultimately, the co-creation of knowledge that is more likely to be valued by members of the research community and by those the research is meant to benefit.

#### The co-creation of knowledge: convergence upon a common aim

CBPR and IKT are used to evoke engagement within and between researchers and knowledge users in partnerships to foster mutually informing and bi-directional exchange of information and to promote shared learning and the co-creation of knowledge [36, 80]. Researchers employing CBPR or IKT report that co-created knowledge is more likely to be used in health systems by knowledge users [19, 56]. The existence of partnerships between knowledge users and researchers serves as a catalyst for increasing both the use and the relevance of the research [17]. Nevertheless, there is an ongoing challenge for researchers to understand how and what types of partnership and participation best enhance the development, uptake, and use of research knowledge [52, 74]. IKT and CBPR users aim to accommodate and facilitate the engagement and involvement of researchers with knowledge users to co-create and apply knowledge, and they can both contribute evidence to existing implementation practices for collaborative research.

Public involvement in research is situated in a changing environment, and researchers are seeking guidance on how to consider and operationalize knowledge user involvement and engagement. The development of knowledge for use in health systems, like all science, is recognized as a values-laden process [81]. CBPR and IKT are approaches that can contribute to consideration of what constitutes knowledge through the incorporation of knowledge user expertise with that of the researcher. As research approaches, they can each foster opportunities for people who are affected by the research to have a say in what and how publicly funded research is undertaken [82].

CBPR and IKT have been developed within distinct research traditions, but both have demonstrated success with an array of knowledge user–researcher collaborations and thus bring unique strengths to bear upon an aim that is valued [65, 67] and commonly held: specifically, the creation of knowledge that is the result of both researcher and knowledge user expertise. For this reason, we urge consideration of both CBPR and IKT approaches and processes when designing and conducting a collaborative research study that has co-creation of knowledge as the aim. Further, both CBPR and IKT lead to the generation of outcomes that can be characterized by the precepts of “knowledge democracy,” where knowledge is defined as “Facts, information, and skills acquired through experience or education; the theoretical or practical understanding of a subject” [83] and democracy as “The practice or principles of social equality” [84] and together contribute to advance the implementation science and practices for collaborative research.

## Conclusion

There is growing interest and continued effort to develop and use collaborative approaches to generate knowledge between knowledge users and researchers for use in health systems. While CBPR and IKT have distinct origins and practices, they share the aim of fostering democratic processes in the co-creation of knowledge. Better understandings of CBPR and IKT will enable researchers and knowledge user partners to appropriately use and leverage knowledge from each approach. CBPR and IKT create opportunities to challenge assumptions about for whom, how, and what is defined as knowledge and to develop and integrate research findings into health systems. We urge consideration of CBPR and/or IKT approaches and processes in the design and conduct of research studies to advance implementation science and its practice for the conduct of collaborative health systems research.
